# Identification and validation of differentially expressed genes in intramuscular fat metabolism in *Guizhou yellow chickens* using RNA-Seq analysis

**DOI:** 10.1371/journal.pone.0326128

**Published:** 2025-06-16

**Authors:** Yingping Tian, Xiaoya Wang, Yongchao Rao, Xiaohong Zhou, Yaozhou Jiang, Qinsong Liu, Sheng Wu, Fuping Zhang

**Affiliations:** 1 Key Laboratory of Animal Genetics, Breeding and Reproduction in the Plateau Mountainous Region, Ministry of Education, Guizhou University, Guiyang, Guizhou, China; 2 College of Animal Science, Guizhou University, Guiyang, Guizhou, China; 3 Qianxi City Agriculture and Rural Bureau, Qianxi, Guizhou, China; 4 Guizhou Institute of Biology, Guiyang, Guizhou, China; University of Hawai'i at Manoa, UNITED STATES OF AMERICA

## Abstract

Intramuscular fat (IMF) content is crucial for meat quality, and genetic, environmental, and nutritional factors influence its deposition. This study aims to identify genes involved in the regulation of IMF deposition in *Guizhou Yellow Chickens*. Thirty *Guizhou Yellow Chicken* hens aged 120 days were selected, and their IMF content was measured using the Soxhlet extraction method. The IMF content was divided into a high IMF group (H group, n = 4) and a low IMF group (L group, n = 4). RNA-seq was performed on the H and L groups to screen out signaling pathways and key genes that affect IMF deposition. A total of 259 differentially expressed genes(DEGs) were screened, including 195 that were up-regulated and 64 that were down-regulated. Critical genes such as *COL1A1*, *COL1A2*, *COL6A3, PLTP*, *LPIN1*, *ITGA8,* and *FN1* were identified as key influencers of IMF deposition in *Guizhou Yellow Chickens*. The slow virus interference vector has demonstrated that interfering with *COL1A1* can inhibit the proliferation ability of chicken preadipocytes and reduce lipid droplet accumulation. In addition, interference with *COL1A1* significantly inhibited the expression of *PLTP*, *ABHD6*, *LPIN1*, and *PTGS2* and decreased the levels of triglycerides and cholesterol at 4 and 8 days. The results further showed that *COL1A1* is a key gene in the gene regulatory network during fat deposition, and the interaction of these genes affects the proliferation and differentiation of fat cells, thereby reducing the accumulation of fat droplets in muscle fibers. This study indicates that the *COL1A1* gene is associated with IMF formation in *Guizhou Yellow Chickens*, providing a critical gene for selecting markers to control IMF formation and laying a foundation for future poultry meat quality breeding.

## Introduction

Chicken meat is high in protein, low in calories, and low in cholesterol, making it a primary source of high-quality animal protein for humans. Consequently, broiler breeding has become a key area of focus in animal husbandry research. However, with social and economic development and rising living standards, consumers are increasingly demanding meat quality. IMF is a critical factor in meat quality, influencing attributes such as tenderness, water retention, shear force, and flavour [1]. The number of adipocytes and their lipid deposition capacity are closely related to the IMF content. In animals, the number of adipocytes is determined before birth, while lipid deposition capacity is influenced by various factors after birth. Moderately increasing IMF content enhances meat flavor, juiciness, and palatability, contributing to improved meat quality and the water-holding capacity of meat products. Additionally, higher IMF content can improve poultry meat quality by reducing drip loss and cooking loss [2].

Current broiler farming has mainly concentrated on augmenting lean meat output, expediting growth rates, and promoting the development of breast and leg muscles. This has resulted in a decrease in IMF content, thereby reducing the acceptability of the meat for consumers [3]. In chicken meat, the IMF content increases with age, promoting improvements in flavor and texture [4]. The heritability of IMF in chickens ranges from 0.21 to 0.81, and studies have shown that genetic breeding is an effective method to increase IMF content in chickens [5]. However, in practical production, IMF content can only be measured post-slaughter, posing challenges in breeding. Therefore, the use of molecular marker-assisted selection serves as an effective approach to enhance IMF content in livestock and poultry [6]. Numerous studies have shown that certain functional candidate genes can regulate the process of IMF deposition. For instance, the perilipin-1 (*PLIN1*) gene is significantly enriched in the peroxisome proliferator-activated receptor (PPAR) signaling pathway associated with lipid metabolism, with its expression level being markedly higher in pigs with high IMF content compared to those with low IMF content. Furthermore, *PLIN1* gene knockout reduces triglyceride content and lipid droplet size in pig adipocytes [7]. As a key inhibitor of matrix metalloproteinases (MMPs), *TIMP2* can regulate intramuscular fat deposition in chickens through the ECM-receptor interaction pathway under the influence of muscle satellite cells [8]. *FABPs* are involved in the uptake, intracellular metabolism, and transport of long-chain fatty acids, and all members of the *FABP* family are considered to be closely related to IMF deposition. Among them, *H-FABP* and *A-FABP* have been widely demonstrated as key genes influencing IMF in animals such as sheep, chickens, and pigs [9–11].

Despite the identification of genes associated with intramuscular fat deposition, the content of IMF is a quantitative trait governed by numerous genes with minor effects. A single or a few genetic markers are inadequate to precisely elucidate the genetic foundation of this trait. Furthermore, these marker loci are subject to variations across different breeds, complicating their application in breeding programs. In recent years, the rapid advancement of high-throughput technologies has enabled transcriptome sequencing (RNA-seq) to emerge as a robust molecular tool for comparing gene expression profiles at the transcriptome level across various physiological states, developmental stages, or environmental conditions. This allows researchers to screen out genes that show significant changes in expression [12]. Therefore, transcriptomics is widely recognized as an effective method to directly identify candidate genes and regulatory mechanisms associated with IMF deposition at the genomic level [13]. Li et al. performed RNA-seq on the breast muscle of *Beijing-You chickens* at different developmental stages and identified several DEGs related to energy metabolism, including *ACOT9*, *CETP*, *LPIN1*, *DGAT2*, *RBP7*, *FBP1*, and *PHKA1*. These genes were found to potentially regulate IMF deposition [14]. Genes such as *L3MBTL1*, *TNIP1*, *HAT1*, and *BEND6* were also identified by RNA-seq as significantly positively correlated with high IMF and significantly negatively related to the low AFW, which could be relevant biomarkers for chicken breeding [15].

*Guizhou Yellow Chicken* is a hybrid breed from the cross of *New Hampshire*, *Plymouth Rock*, and *Weining chickens*. It is highly adaptable to the climate of the Yunnan-Guizhou Plateau. The breed is characterized by a high feed conversion rate, rapid growth, abundant egg production, and bright feather coloration. Its meat is known for being fresh and waxy, making it an ideal ingredient for the renowned Guizhou dish, “Chicken with Chilies,” which is loved by many consumers. To increase the IMF content of *Guizhou yellow chicken*, this study analyzed RNA-seq data of the chest muscle of *Guizhou Yellow Chickens* with significant differences in IMF content to explore the regulatory mechanism of IMF metabolism and identify candidate genes affecting IMF deposition. A lentivirus interference vector was used to verify its function and explore its effect on adipocyte differentiation and its potential molecular mechanism. This research aims to provide a foundational basis and additional references for enhancing meat quality in poultry breeding and producing high-quality chicken meat.

## Materials and methods

### Ethics approval

All work in this experiment follows the Chinese Animal Welfare Guidelines and has been approved by the Subcommittee of Experimental Animal Ethics Guizhou University (Guiyang, People’s Republic of China) with approval number EAE-GZU-2022-E054.

### Test material

The research chicken farm at Guizhou University provided the experimental animals for this study. *Guizhou Yellow Chickens* were raised from 1 to 120 days of age under the same environment and nutritional conditions, with free access to food and water. Thirty *Guizhou Yellow Chickens* of comparable body weight at 120 days of age were selected and subjected to slaughter via the carotid bleeding method following a 12-hour fasting period. The left pectoralis muscle was meticulously excised, and 2g samples were collected in RNase-free tubes. The remaining portion of the pectoralis muscle was appropriately labeled, sealed, and stored at −80°C for subsequent RNA-seq and IMF analysis. Additionally, abdominal adipose tissue from 10-day-old broilers was collected as the preadipocyte culture material.

### IMF analyze and transcriptome sequencing

IMF content of *Guizhou Yellow Chicken* breast muscle was determined by utilizing the Soxhlet extraction method, according to the national standard GB5009.6−2016. In the study, highly significant differences in IMF content among individuals prompted the selection of two distinct groups: the high IMF group (H group, comprising four animals) and the low IMF group (L group, also consisting of four animals). Total RNA was extracted from the pectoral muscle of the H and L groups using a Trizol (Invitrogen, Carlsbad, USA) reagent according to the instructions of manufacturer. The RNA concentration and purity were measured using a micro UV-Vis spectrophotometer from Thermo Fisher Scientific. Subsequently passing quality control, libraries were prepared and sequenced on the Illumina platform. Sequencing and bioinformatics analysis were conducted by Qingdao BioMarker Technologies Company.

### Bioinformatics analysis of sequencing data

To ensure data quality, sequences containing ligations and low-quality reads were removed from the sequencing data (Including Reads that remove >10% of N and reads that remove more than 50% of the entire read for bases with a quality value of Q ≤ 10). The Q30 and GC content of the remaining valid data were calculated. The alignment software HISAT2 was used to align the clean reads to the chicken reference genome (version: GRCg6a_NCBI), and the comparison flowchart can be found in S1 Fig.

Fragments per kilobase of transcript per million mapped reads (FPKM) were used as an index to calculate the expression level of each transcript in every library using the Cuffdiff program. The expression levels of the H and L groups were statistically analyzed using DESeq software (version 1.6.3). DEGs with significantly different expression levels between the two groups were identified based on the screening criteria of fold change ≥ 1.5 and *P* < 0.05. Pearson’s correlation coefficient was employed to assess the repeatability of samples both within and between groups [16], thereby eliminating expression differences attributed to biological variability [17,18]. The Gene Ontology (GO) database was employed for GO functional analysis of DEGs. *P*-values were corrected for false discovery rate (FDR), and enrichment was considered significant when FDR was ≤ 0.05. The KOBAS 2.0 software was employed to detect significant pathways associated with DEGs via the Kyoto Encyclopedia of Genes and Genomes (KEGG) pathway analysis. The screening condition for DEGs in pathways was set as *P* ≤ 0.05.

### QRT-PCR test

Six DEGs were randomly selected from the NCBI database (http://www.ncbi.nlm.nih.gov) based on the published mRNA sequence. The qRT-PCR primers were designed using Primer Premier 5 software and synthesized by Beijing Qingke Biotechnology Company (S1 Table). The RNA was reverse transcribed to cDNA using a Reverse Transcription Kit (Thermo Fisher, Shanghai), which confirmed the utilization of gel electrophoresis. Subsequently, quantitative PCR (qPCR) was performed using a Fluorescent PCR Kit (Thermo Fisher, Shanghai) according to the instructions of manufacturer. The reaction system (20 μL) included 10 μL of SYBR Green qPCR Mix, 0.5 μL each of upstream and downstream primers (10 μmol/L), 1 μL of cDNA, and 8 μL of double-distilled water. The PCR reaction conditions comprised predenaturation at 95°C for 2 min and denaturation at 95°C for 15 s, annealing at 60°C for 30 s, and extended to 72°C for 30 s over 40 cycles. Additionally, the default settings were utilized for the dissolving curve parameters while β-actin was employed as the internal reference gene, and gene expression was calculated using the 2^-ΔΔCt^ formula. Analysis of each sample was repeated three times, with the SD of the threshold (Ct) value set at ≤0.5.

To understand the interaction between genes, qRT-PCR was used to detect the expression of *PLTP, ABHD6, LAMA3, LPIN1,* and *PTGS2* genes related to fat deposition when the *COL1A1* gene was interfered with. Primer information was provided by Anhui General Biol company synthesized primers (S2 Table).

### Preadipocyte culture and differentiation

Abdominal fat was collected from 10-day *Guizhou Yellow chicken* under aseptic conditions. The fat was rinsed with PBS containing 2% double antibodies and placed in sterilized petri dishes. The fat was then cut into small pieces and digested in a type I collagenase solution at 37°C for 65 min. After digestion, the solution was neutralized with an equal volume of complete medium [Dulbecco’s Modified Eagle Medium (DMEM)/F12 + 10% fetal bovine serum (FBS) + 100 IU/mL penicillin]. The digested solution was filtered through a 400-mesh sieve to remove undigested tissue and debris, and the filtrate was centrifuged at 1600 γ/min for 10 min. The supernatant was discarded, and the precipitate was rinsed with a complete medium and centrifuged again at 1600 γ/min for 10 min. The resulting precipitate was resuspended in a complete medium and incubated at 37°C with 5% CO2. The cells were then incubated for an hour, the medium was discarded, and the cells were washed twice with PBS to obtain preadipocytes. Finally, the medium was added to the complete medium and cultured, with the medium being changed every 1 or 2 days.

### Lentivirus transduction

The results of RNA-seq showed that genes such as *COL1A1*, *COL1A2*, *CAPN2*, *LPIN1*, *PLTP*, and others involved in focal adhesion, ECM-receptor interaction, TGF-β and PPAR signalling pathways may be the key roles affecting IMF deposition in *Guizhou Yellow Chickens*. Among them, *the COL1A1* gene has been identified as a candidate gene for IMF, and its role has been confirmed by many studies [19–25]. However, the lack of studies on knocking down the *COL1A1* gene to explore its molecular mechanics significantly increases our research interest in *COL1A1* gene. Therefore, in this study Genepharma Designer3.0 was used to design the RNA interference sequence of the chicken *COL1A1* gene (CAGCCAACAGATCGAGAACAT). The interference sequence was synthesised by Gemma Suzhou. Human embryonic kidney cells (293T) were cultured in a T25 flask until they reached a concordance of about 80–90 percent. Subsequently, recombinant shuttle plasmid and package plasmid containing target sequence were transfected into 293T cells using RNAi-Mate transfection reagent according to the instructions of the manufacturer. Finally, lentivirus overexpressing *COL1A1* (sh-*COL1A1*) was obtained, and blank vector control lentivirus was prepared. Furthermore, chicken preadipose cells were transfected using the Lipofectamine 2000 reagent. The transfected chicken preadipocyte suspension was inoculated in a 6-well plate and incubated in an incubator at 37°C with 5% CO2 for 48 hours. After washing twice with a PBS solution containing double antibodies, the medium was replaced with an induced differentiation medium (10% FBS + 1 μmol/L dexamethasone + 5 μg/mL insulin + 0.5 mmol/L IBMX) and labelled Day 0. After 2 days of incubation, the medium was replaced with a maintenance differentiation medium (10% FBS + 1 μg/mL insulin). In addition, the medium was changed every 2 days.

### Oil Red O staining and quantitative analysis

Following virus infection and differentiation induction, cells were removed from the medium on Day 4 and Day 8 of differentiation. They were then washed three times with PBS and fixed with 4% paraformaldehyde for 30 minutes, then washed three more times with PBS (5 minutes each wash) and permeabilized with 60% isopropanol. Moreover, the Oil Red O staining solution was added to cover the bottom of the cell culture plate completely and stain the cells for 10 min to 30 min. The staining solution was removed, and the cells were washed three times with PBS for 5 min per wash. Subsequently, hematoxylin staining was performed for 1 min, and the cells were washed three times with PBS for 5 min per wash. An appropriate amount of PBS was added to preserve lipid droplets, and images were promptly captured using a fluorescent inverted microscope.

### Triglyceride and cholesterol measurement

IMF refers to the fat content within muscle tissue, primarily composed of triglycerides (TG) but also encompassing phospholipids (FLIP) and cholesterol (CH). IMF is a complex trait, thus identifying the key genes involved in its deposition presents significant challenges. The analysis of TG, FLIP, and CH can facilitate a more streamlined phenotype characterization and aid in uncovering the mechanisms underlying fat deposition [26,27]. In this study, the detection of TG and CH was performed to reflect changes in lipid levels. Triglyceride and cholesterol concentrations in the medium were quantified using kits on Days 4 and 8 of *COL1A1* interference-induced differentiation. Experiments were conducted following the operating instructions of the Glycerol Test Kit and Cholesterol Test Kit provided by Shanghai EnzymeLink Biologicals.

### Statistics and data analysis

Data were processed using Excel 2017 for statistical analysis. The expression levels of fat deposition-related genes and the internal reference gene *GAPDH* in chicken preadipocytes were determined using the 2^-ΔΔCt^ method. The results were presented as means ± SD. To compare gene expression levels between groups, an independent samples t-test was performed using SPSS version 20 software, while Graphpad Prism 8.0 was used for data visualization.

## Results

### RNA-seq data analysis

As shown in Table 1, thirty *Guizhou Yellow Chickens* were selected and categorized into two groups based on their IMF content, which included a high-IMF group (n = 4) and a low-IMF group (n = 4). The high-IMF group (Group H) had an average IMF content of 2.10% ± 0.29%, which was significantly higher (*P* < 0.01) than the average IMF content in the low-IMF group (Group L), measured at 1.42% ± 0.13%. RNA-seq was conducted on the high-IMF and low-IMF groups using the Illumina platform to investigate the differential genes influencing IMF content in *Guizhou Yellow Chickens*. After quality control, the sequencing produced 5.427 GB of clean data, as detailed in Table 2. Each sample had an average GC content of 52.62%, and the Q30 score was > 92.25%. The comparison rate of the sequencing reads relative to the chicken reference genome was > 82.27%, indicating high sequencing accuracy, while the comparison rate relative to the genome’s exons was 88.33%, further confirming the reliability and quality of the data for subsequent analyses.

**Table 1:** Analysis of IMF content in breast muscle of *Guizhou Yellow Chicken.*

Index	L group (n = 4)	H group (n = 4)	*P*-value
Weight/g	1715.30 ± 114.07	1762.30 ± 126.30	0.311
IMF/%	1.42 ± 0.13^B^	2.10 ± 0.29^A^	<0.01

In the same row, values with no letter or the same letter superscripts mean no significant difference (*P* > 0.05), while with different small letter superscripts mean significant difference (*P* < 0.05), and with different capital letter superscripts mean significant difference (*P* < 0.01).

**Table 2:** mRNA sequencing data and comparison efficiency statistics.

Sample	Clean reads	Clean bases	GC content	Q30/%	Mapped ratio	Mapped to exon
L1	19774289	5906435822	52.17	94.56	86.82	88.39
L2	24379457	7285884300	52.51	92.99	85.42	88.25
L3	23989251	7177118522	52.64	93.4	85.09	88.12
L4	26381485	7875559976	53.2	92.25	82.27	88.23
H1	22784719	6816666722	52.91	93.07	84.5	88.44
H2	20653445	6173822370	53.35	93.11	86	88.49
H3	23211835	6945403672	52.24	93.15	85.12	87.89
H4	20412849	6092488420	51.97	93.78	84.7	88.81
Average	181587330	6784172476	52.62	93.29	84.99	88.33

H1 ~ 4 and L1 ~ 4 represent high and low IMF 4 biological replicates of *Guizhou Yellow Chicken*, respectively; Q30, Ratio of bases with mass value greater than 30 to total bases.

### Differential expression analysis and qRT-PCR validation

**Fig 1:**
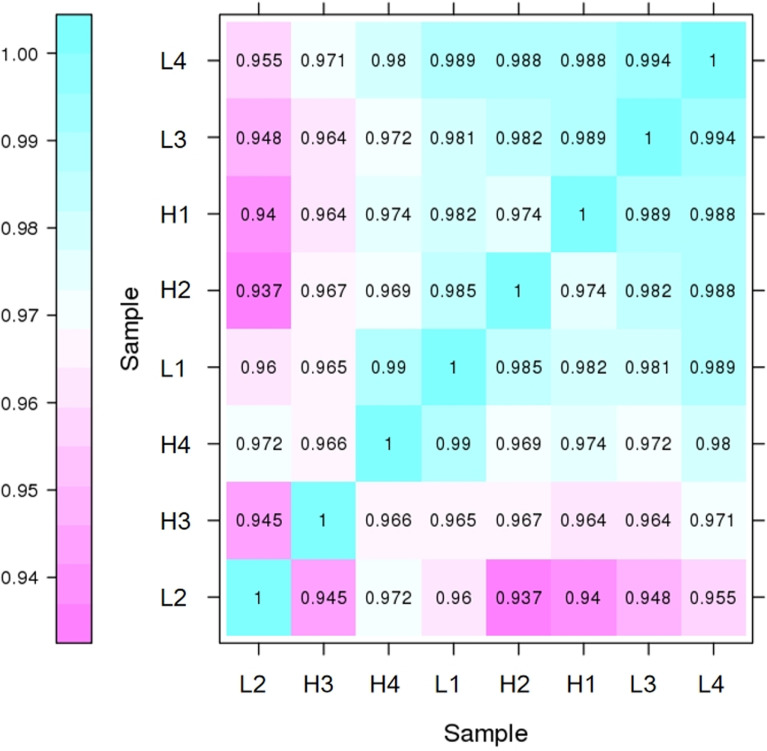
Pearson’s correlation analysis of chest muscle samples of *Guizhou Yellow Chicken* between group L and group H.

**Fig 2:**
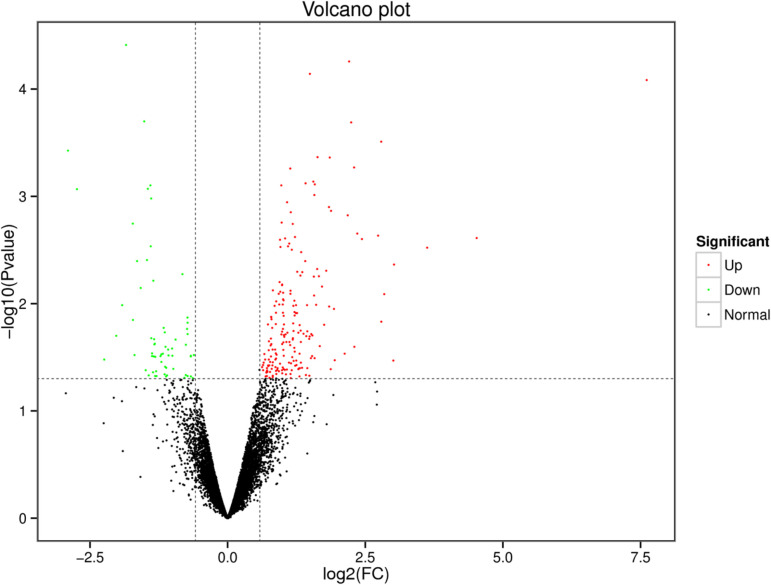
Volcano plot displaying DEGs. Green dots represent down-regulated DEGs, red dots represent up-regulated DEGs, and black dots represent non-DEGs.

**Fig 3:**
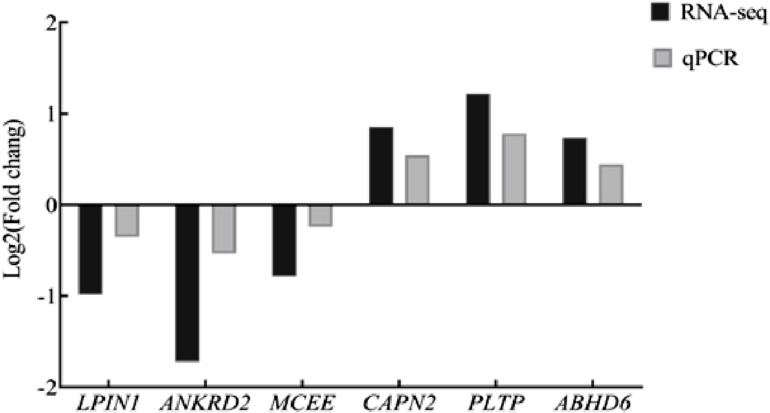
The differentially expressed genes were confirmedby qPCR.

Fig 1 shows the correlation between the gene expression levels of biological replicates within each group. The R-values for the four replicates in groups L and H were all > 0.9, indicating the reproducibility of the samples and the absence of significant biological variability [referencing Materials and Methods, reference. EdgeR [28] was used to identify the DEGs in groups H and L. A total of 259 DEGs were identified between the two groups, with 64 genes up-regulated and 195 down-regulated (Fig 2 and S3 Table). To validate the RNA-seq data of the H and L IMF groups in *Guizhou Yellow Chicken*, a subset of DEGs was randomly selected including up-regulated (*CAPN2*, *PLTP*, and *ABHD6*) and down-regulated genes (*LPIN1*, *ANKRD2*, and *MCEE*). RT-qPCR was performed to measure the expression of these six genes in pectoral muscle tissue. The qPCR validation results (Fig 3) revealed a consistent expression trend for these DEGs in the H and L IMF groups, confirming the reliability and reproducibility of the RNA-seq data.

### GO enrichment and KEGG pathway analysis

**Fig 4:**
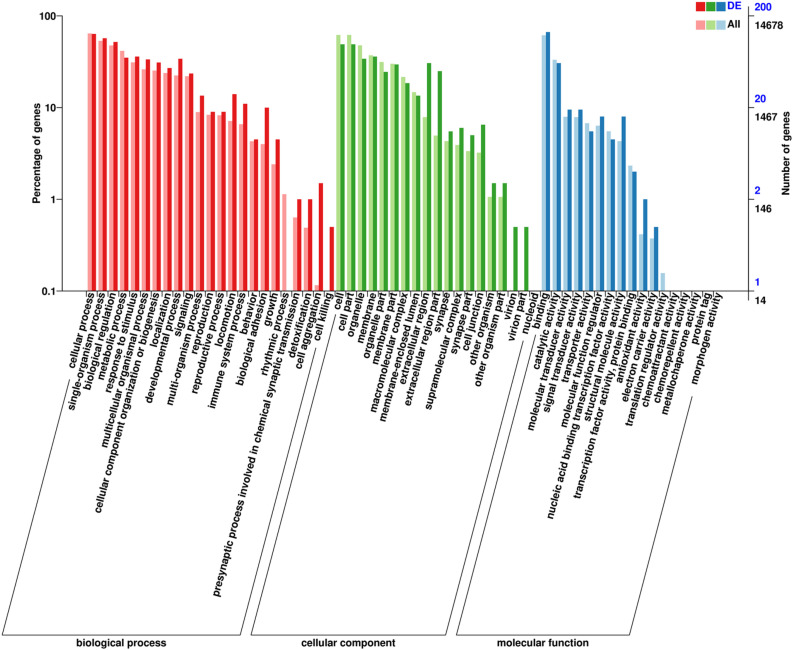
The most enriched GO terms of differentially genes. X-axis represents GO terms and classifications; Y-axis represents number of DEGs annotated to the term (right) and percentage of that over total annotated genes (left).

**Fig 5:**
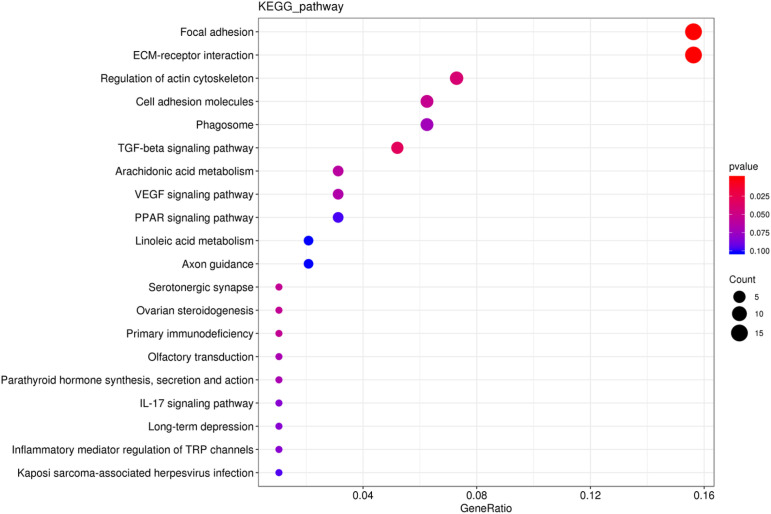
KEGG enrichment analysis of DEGs revealed the top 20 pathways identified through enrichment. The size of the dots indicates the number of expressed genes within each pathway, while the color of the dots represents the *P*-values of the significantly enriched pathways.

The functionality of 200 DEGs was analyzed through GO. The results show that most DEGs are primarily involved in binding, metabolic processes, biological regulation, responses to stimuli, catalytic activities, and cellular regulation of processes (Fig 4 and S4 Table). These DEGs may directly or indirectly participate in fat formation, such as lipoprotein metabolism, triglyceride synthesis, lipid oxidation and stress regulation of fat energy storage. KEGG pathway analysis demonstrated the top 20 pathways exhibiting the lowest *P*-values, which are illustrated in the scatter plots in Fig 5. DEGs showed significant enrichment in pathways related to focal adhesion and ECM-receptor interactions. Furthermore, notable enrichment was found in pathways associated with the regulation of actin cytoskeleton, cell adhesion molecules, and the TGF-β signaling pathway. These pathways are likely implicated in fat metabolism and IMF deposition. Additionally, DEGs (including *LAMA3*, *CHAD*, *COL1A1*, *COL1A2*, COL6A1, *COMP*, *FN1*, and *ITGA8*) were enriched within ECM-receptor interaction and focal adhesion. Several pathways related to lipid deposition, including arachidonic acid metabolism, PPAR signaling pathway, and linoleic acid metabolic pathways, showed varying degrees of enrichment. (Table 3 and S2 Fig).

**Table 3:** Some of the KEGG enrichment pathways associated with fat deposition.

Code	KEGG pathway	Down-regulated gene	Up-regulated gene
1	Focal adhesion	*LAMA3*	*CAPN2,CHAD,COL1A1,COL1A2* *COL6A1,COL6A2,COL6A3,COMP* *FN1,ITGA11,ITGA8*
2	ECM−receptor interaction	*LAMA3*	*CHAD,COL1A1,COL1A2* *COL6A1,COL6A2,COL6A3,COMP,* *FN1,ITGA11,ITGA8,LOC101748153,THBS4,TNX*
3	Regulation of actin cytoskeleton	*ITGA11,ITGA8,SCIN*	*LOC101748153,THBS4,TNX* *CXCL12,ENAH,FN1*
4	Cell adhesion molecules	*CD2,CNTNAP1,HHLA2*	*ITGA8,MHCIA2,SELPLG*
5	TGF−beta signaling pathway	*FBN1,FMOD,FST,PLIN1*	*SMAD7,SMAD7B*
6	PPAR signaling pathway	–	*C1QTNF2,PKIB,PLTP*

*LAMA3*, laminin subunit alpha 3; *CAPN2*, Calproteinase 2; *CHAD*, Chondroadherin; *COL1A1*, Collagen type I alpha 1 chain; *COL1A2*, Collagen type I alpha 2 chain; *COL6A1*, Collagen type VI alpha 1 chain; *COL6A2*, Collagen type VI alpha 2 chain; *COL6A3*, Collagen type VI alpha 3 chain; *COMP*, Cartilage oligomeric matrix protein; *FN1*, Fibronectin 1; *ITGA11*, Integrin subunit alpha 11; *ITGA8*, Integrin subunit alpha 8; *LOC101748153*, Laminin subunit beta-2-like; *THBS4*, Thrombospondin 4; *TNX*, Tenascin-X; *CXCL12*, C-X-C motif chemokine ligand 12; *ENAH*, ENAH actin regulator; *SCIN*, Scinderin; *CD2*, Cutin deficient 2; *CNTNAP1*, Contactin associated protein 1; *HHLA2*, HERV-H LTR-associating 2; *MHCIA2*, Major histocompatibility complex Y; *SELPLG*, Selectin P ligand; *FBN1*, Fibrillin 1; *FMOD*, Fibromodulin; *FST*, Follistatin; *SMAD*, SMADs family member; *C1QTNF2*, C1Q and Tumor necrosis factor related 2; *PKIB*, Protein kinase inhibitor beta; *PLTP*, Phospholipid transfer protein; *PTGS2* : Prostaglandin-endoperoxide synthase 2.

### Sh*-COL1A1* interference efficiency test

**Fig 6:**
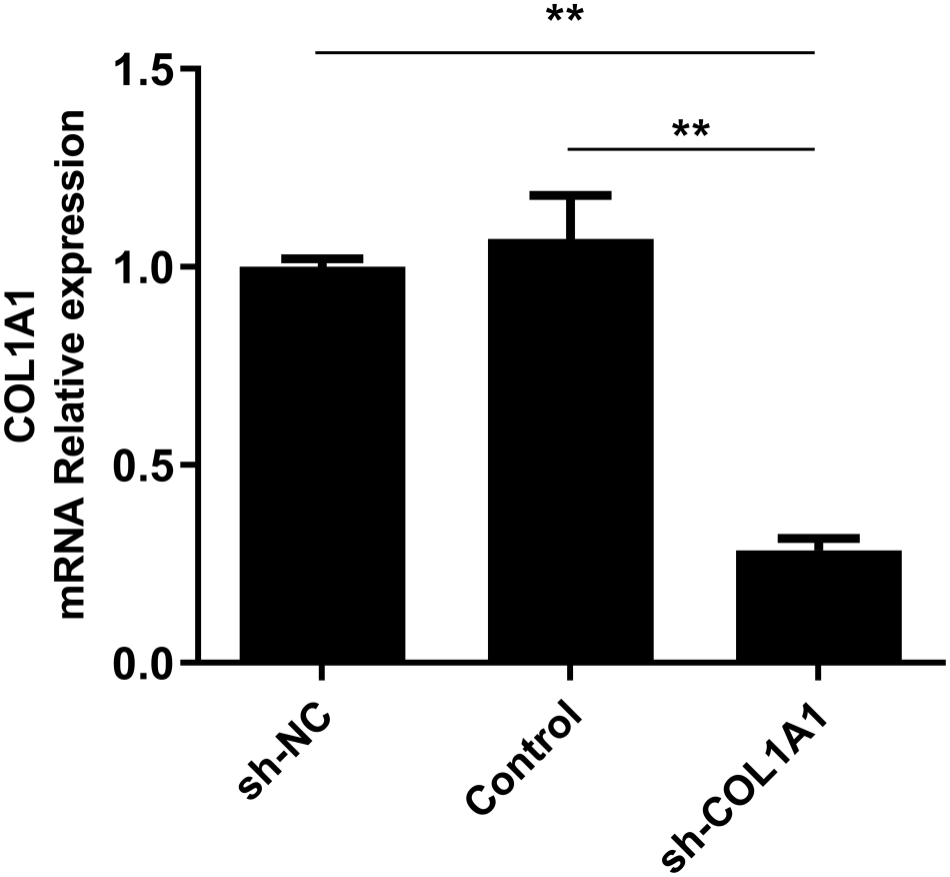
qRT-PCR validation of *COL1A1* gene interference efficiency.

A lentivirus vector system was used to interfere with the *COL1A1* gene by expressing short hairpin RNAs (shRNAs). In order to further determine the interference efficiency of the *COL1A1* gene, the NC group was used as a calibration, and *GAPDH* was used as the internal reference gene. After 72h inoculation of chicken intramuscular adipocytes, total RNA was extracted, and qPCR was performed. As shown in Fig 6, compared with the NC group, the interference efficiency of the *COL1A1* gene reached 72%, indicating that the interference efficiency was satisfactory.

### Effect of *COL1A1* gene interference on proadipocyte proliferation and adipocyte differentiation

**Fig 7:**
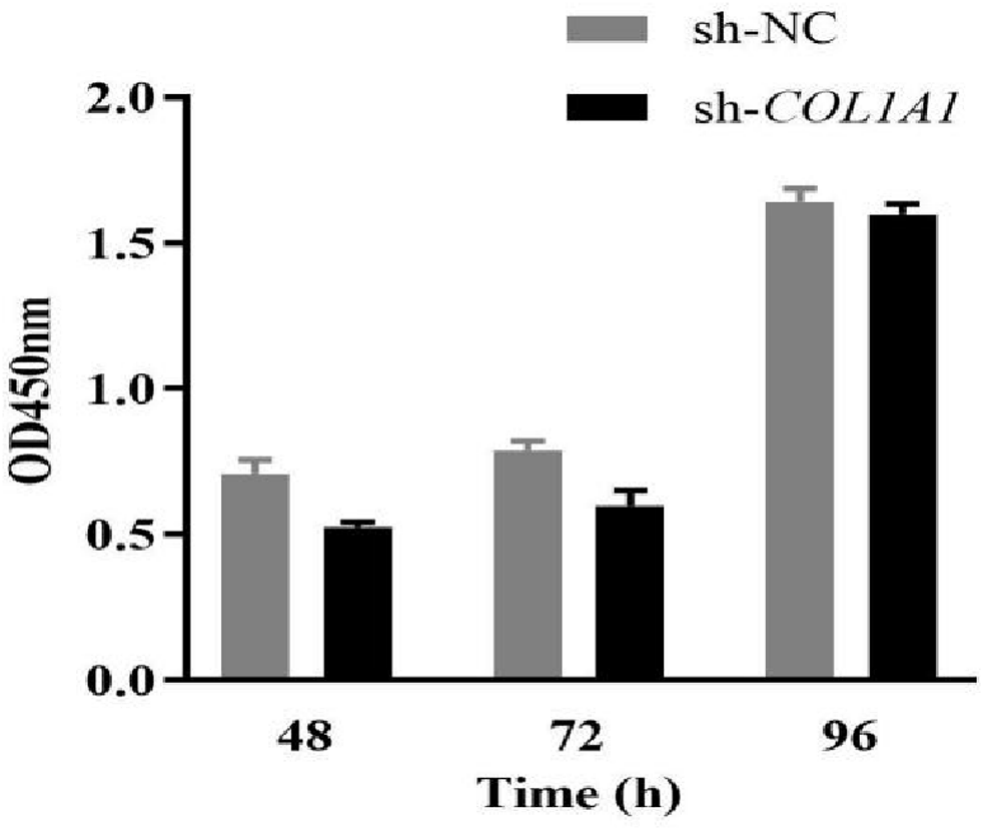
The effect of *COL1A1* interference on proliferation of chicken preadipocytes.

Chicken preadipocytes were transfected with the *COL1A1* interfering vector and the NC group when cells reached approximately 80% confluence. Cell proliferation was assessed using a CCK-8 Kit, and the absorbance was measured at 450 nm. Cell growth curves were plotted using time (h) on the x-axis and the measured absorbance values (OD) of different treatment groups at various time points on the y-axis. As shown in (Fig 7), the proliferation capacity of chicken preadipocytes was lower at 48, 72, and 96 h after interference compared with the control group. Suggesting that the COL1A1 genetic factor promotes the proliferative capacity of precursor adipocytes.

**Fig 8:**
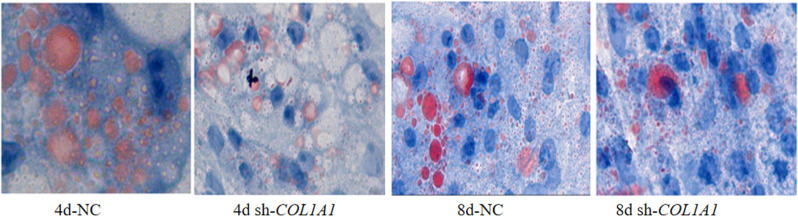
Lipid deposition capacity of preadipocytes during differentiation after *COL1A1* interference (Inverted microscope, 400×).

Oil Red O staining was used to observe changes in adipocyte lipid droplet deposition after lentivirus-mediated interference with sh-*COL1A1* in chicken preadipocytes at two-time points, including Day 4 and Day 8. Cell differentiation and lipid droplet deposition decreased significantly in the *COL1A1*-interference group at both time points compared with the control group, which indicated inhibited lipid droplet accumulation (Fig 8).

### Testing of triglyceride and cholesterol levels

**Fig 9:**
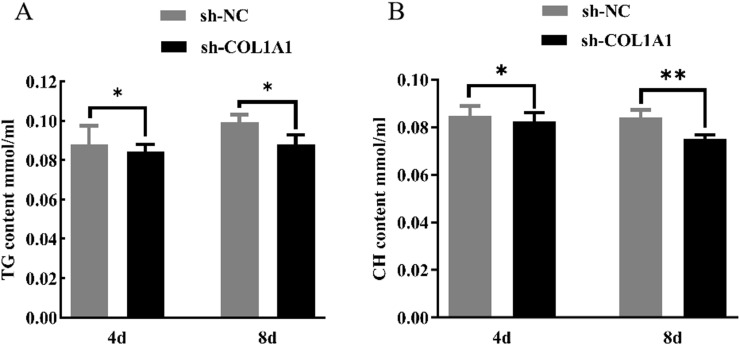
Effect of interfering with the *COL1A1* gene on triglycerides and cholesterol in chicken preadipocytes.

Kits detected triglyceride and cholesterol level changes in adipocytes following *COL1A1* gene interference. As shown in Fig 9A and 9B, the glycerol and cholesterol levels in the medium decreased significantly (*P* < 0.05) after transfection with sh-*COL1A1* on both Day 4 and Day 8 compared with the control group. Moreover, on Day 8, a highly significant decrease (*P* < 0.01) was observed. The results indicated that the formation of COL1A1 gene in adipocytes was inhibited.

### Testing of fat deposition-related genes

**Fig 10:**
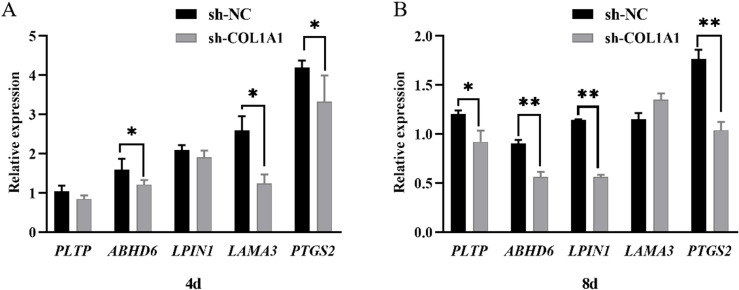
Expression levels of genes related to fat deposition after *COLIAI* gene interference.

In order to study the effect of *COL1A1* gene interference on RNA expression levels of adipose-deposition-related genes, qRT-PCR was used to verify the relative expression levels of *PLTP*, *ABHD6*, *LPIN1*, *LAMA3* and *PTGS2* genes. As can be seen from Fig 10A and 10B, after transfection with the interfering *COL1A1* gene on Day 4 and Day 8, the mRNA expression levels of the *PLTP* and *LPIN1* genes were lower compared to the control group. Additionally, the mRNA expression of the *ABHD6*, *LAMA3*, and *PTGS2* genes was significantly reduced (*P* < 0.05) compared to the control group. After 8 days, the *PLTP* gene expression was significantly lower (*P* < 0.05), while the *ABHD6*, *LPIN1*, and *PTGS2* gene expressions were significantly lower (*P* < 0.01) than those in the control group. These results indicated that interference with the *COL1A1* gene could inhibit the expression level of adipose-related genes.

## Discussion

IMF deposition is a highly complex process that can be affected by genetic, managerial, and nutritional factors. Among these factors, genetics is a significant driver [29]. In this study, to identify candidate genes associated with IMF deposition, 259 DEGs were screened from pectoral muscle tissue samples with significant differences in IMF deposition. These DEGs were analyzed using GO and KEGG pathway analyses. The results revealed that the DEGs *PLTP* and *LPIN1* were enriched in the *PPAR* and TGF-β signaling pathways, respectively, which have been shown to play crucial roles in regulating fat deposition and muscle development in chicken meat breeding. This is consistent with study by Liu L and Cheng J [26,30], which have shown *PPAR* and TGF-β signaling pathways play critical roles in IMF deposition. Similarly, the activation of the transcription factor *PPARG* in the PPAR pathway promotes the interaction between *PLIN1* and *CIDEC*, leading to accelerated lipid droplet formation [31]. During preadipocyte differentiation, *LPIN1* is negatively correlated with PPARγ and C/EBPα expression, promoting the proliferation and differentiation of preadipocytes [32]. Essentially, *LPIN1* functions as a negative regulator of adipogenesis by inhibiting the expression of key differentiation markers. *LPIN1* is consistently highly expressed in adipose tissue, skeletal muscle, and liver, which plays a role in lipid metabolism and energy regulation [33]. In the pituitary and hamstring muscles’ DEGs, the *PLTP* gene participates in the PPAR signaling pathway, affecting lipid metabolism [34]. In this study, *PLTP* was up-regulated in group H vs group L and is known to be highly expressed in adipose tissue, where it regulates glucose and lipid homeostasis and promotes CH efflux [35]. These findings suggest that *PLTP* and *LPIN1* positively regulate fat deposition. Interestingly, the focal adhesion pathway and ECM-receptor interaction were all significantly enriched by genes such as *COL1A1*, *COL1A2*, *COL6A1*, *COL6A2*, *COL6A3*, *CHAD*, *FN1*, and *ITGA8*. Cui Et al. also identified common collagen family genes (*COL6A2*, *COL6A3*, *COL5A2*) in ECM-receptor interactions and plaque adhesion pathways [36], which is similar to our findings. Furthermore, the ECM-receptor interaction and focal adhesion pathways have points of interaction where they play an essential role in maintaining tissue integrity, and these two pathways, along with the PPAR signaling pathway, may contribute to IMF metabolism in chickens [37]. *COL1A1* regulates hormone-induced expression of proteolytic enzymes and clearance of intracellular matrix deposition, suggesting its role in cytoplasmic matrix stabilization [38]. The *COL1A1* gene is up-regulated in IMF cell populations, and both *COL1A1* and *COL1A2* are associated with meat quality traits [39,40]. *COL6A3* plays a role in ECM remodeling and integrin-mediated signaling, impacting IMF deposition [41]. *ITGA8*, a member of the integrin family involved in cell adhesion and ECM signaling, modulates mechanotransduction pathways through its role in integrin-ECM interactions, influencing adipocyte development and lipid storage [42]. *FN1* interactions with integrins, particularly integrin α5β1, facilitate ECM assembly and mechanotransduction, promoting structural support and functional signaling essential for lipid metabolism [43]. In summary, the above evidence suggests that *COL1A1*, *COL1A2*, *COL6A3*, *PLTP*, *LPIN1*, *ITGA8* and *FN1* are key genes involved in IMF deposition in *Guizhou yellow chickens*. Future research will focus on utilizing molecular-assisted breeding techniques to identify and select genes associated with improved IMF content in chickens.

Previous studies on *COL1A1* have primarily focused on human diseases and cancers [44], suggesting that the *COL1A1* gene may serve as a biomarker and therapeutic target for hepatocellular carcinoma and metastasis [45]. *COL1A1* gene was also identified as a key gene in the IMF deposition capacity [19–25], which is consistent with results of this study. However, the mechanism of the *COL1A1* gene affecting the formation and deposition of IMF cells is still unclear, so this gene has generated significant interest for this study. In this study, it was hypothesized that the *COL1A1* gene influences the proliferation and differentiation of preadipocytes and adipocytes, with other genes potentially contributing to this process. The results demonstrated that the knockdown of *COL1A1* reduced the proliferative capacity of chicken preadipocytes at 48, 72, and 96 hours post-intervention. Cell differentiation and lipid droplet formation were notably decreased at 4 and 8 days post-COL1A1 knockdown. These findings indicate that *COL1A1* is a key regulator of adipogenesis, impacting lipid metabolism by modulating the secretion or accumulation of glycerol and CH. The reduced differentiation and lipid accumulation observed could be attributed to the impaired function of fibro/adipogenic progenitors (FAPs), the common progenitor cells from which intramuscular adipocytes and fibroblasts originate [46]. The association between lipid metabolism and ECM remodeling offers insights into the interplay between adipogenesis and fibrogenesis. Adipogenesis and fibrogenesis are interdependent processes, making the role of *COL1A1* crucial in maintaining the balance between these pathways. Furthermore adipose tissue formation is closely linked to the extracellular matrix (ECM), in which *COL1A1* serves as a fundamental component [47]. Disruption of *COL1A1* likely alters the ECM’s structure and function, which may impair the microenvironment necessary for preadipocyte differentiation and lipid deposition [48,49]. *COL1A1* impacts lipid metabolism through structural and signaling roles within the ECM, particularly by interacting with integrin receptors, TGF-β, PPARγ, and Hippo pathways [50,51]. Dysregulation of *COL1A1* expression leads to ECM stiffness, impaired adipogenesis, and disrupted lipid metabolism, contributing to metabolic diseases such as obesity and insulin resistance [52]. The observed reduction in lipid deposition could be explained by a downstream effect of *COL1A1* knockdown on lipid metabolic enzymes or transporters, which deserves further investigation. The findings of this study demonstrate that *COL1A1* knockdown not only impacts the proliferation and differentiation of chicken preadipocytes but also significantly suppresses the expression of several key adipose-related genes, including *PLTP*, *LPIN1*, *ABHD6*, *LAMA3*, and *PTGS2*. The down-regulation of these genes provides insight into the molecular mechanisms by which *COL1A1* influences lipid metabolism and adipogenesis. At both 4 and 8 days post-transfection, the mRNA expression levels of *PLTP* and *LPIN1* were significantly lower in the *COL1A1* knockdown group compared to the control, with *PLTP* showing particularly pronounced suppression at 8 days (*P* < 0.05). These genes play crucial roles in lipid metabolism; *PLTP* is involved in lipid transport and remodeling [53], while *LPIN1* is a key regulator of TG synthesis and adipocyte differentiation [54]. The suppression of these genes suggests that the disruption of *COL1A1* impairs critical pathways necessary for lipid storage and adipocyte function. Furthermore, the mRNA expression levels of *ABHD6*, *LAMA3*, and *PTGS2* were significantly reduced after *COL1A1* knockdown. Notably, at 8 days post-transfection, *ABHD6*, *LPIN1*, and *PTGS2* exhibited highly significant down-regulation (*P* < 0.01), indicating a cumulative effect over time. *ABHD6* plays a role in lipid hydrolysis, contributing to lipid droplet turnover [55], while *PTGS2* (also known as COX-2) is involved in inflammation and lipid metabolism [56]. *LAMA3*, a laminin family member, is critical for ECM integrity and cellular interactions [57]. The suppression of these genes highlights the interconnected roles of ECM organization, lipid metabolism, and cellular signaling in adipogenesis. These results further reinforce the concept that adipogenesis and fibrogenesis are intricately linked processes. As *COL1A1* is essential for the formation and maintenance of the ECM, its knockdown likely disrupts the microenvironment required for the proper expression of adipose-related genes. This aligns with our earlier findings that *COL1A1* knockdown reduces adipocyte proliferation, differentiation, and lipid droplet accumulation, collectively contributing to impaired adipose tissue formation. In summary, the significant down-regulation of adipose-related genes following *COL1A1* knockdown indicates that this gene is a central regulator of adipogenesis and lipid metabolism in chicken adipocytes. These findings provide valuable insights into the molecular mechanisms governing IMF deposition and highlight potential genetic targets for improving meat quality. Future research should focus on elucidating the upstream regulators and downstream pathways of *COL1A1* to clarify further its role in adipocyte biology and meat production traits.

## Conclusions

In this study, candidate genes affecting IMF were identified as *COL1A1*, *COL1A2*, *COL6A3*, *PLTP*, *LPIN1*, *ITGA8* and *FN1* by RNA-seq analysis of high and low IMF groups of *Guizhou yellow chickens*. Further investigation revealed that *COL1A1* is a positive regulator of chicken adipocyte proliferation and differentiation. Moreover, reduced *COL1A1* expression may decrease IMF content via the ECM−receptor interaction and Focal adhesion signaling pathways. These findings provide a theoretical basis for elucidating the molecular regulatory mechanisms of IMF in *Guizhou Yellow chickens* and offer valuable insights for developing genetic improvement and molecular breeding strategies in poultry.

## Supporting information

S1 Fig Flow chart of sequencing data comparison. (PNG)

S2 Fig KEGG enrichment network map of DEGs. The color of the edge represents different pathways, and the color of the gene node represents the difference multiple. The larger the pathway node is, the more genes are enriched into the pathway.
(PNG)

S1 Table Primer information table for 6 genes for qPCR validation. (XLS)

S2 Table 6 DEGs primer information tables related to IMF content. (XLS)

S3 Table DEGs information in H and L groups of *Guizhou yellow chickens.* (XLSX)

S4 Table Gene ontology (GO) term for DEGs. (XLSX)
