# Supplementary figures and images for: Identification and validation of differentially expressed genes in intramuscular fat metabolism in Guizhou yellow chickens using RNA-Seq analysis
Source: PLoS One. 2025 Jun 16;20(6):e0326128. doi: 10.1371/journal.pone.0326128 (PMC12169570; doi:10.1371/journal.pone.0326128)

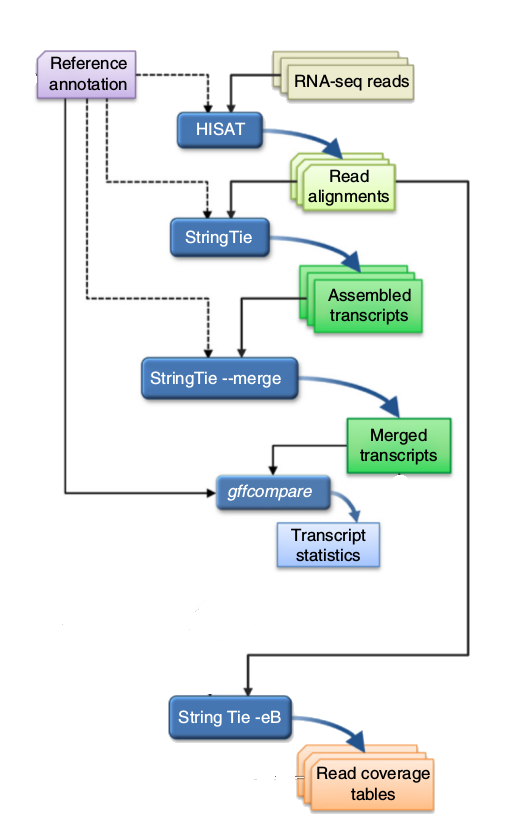

Supplement: S1 Fig — (PNG) [file pone.0326128.s001.png]

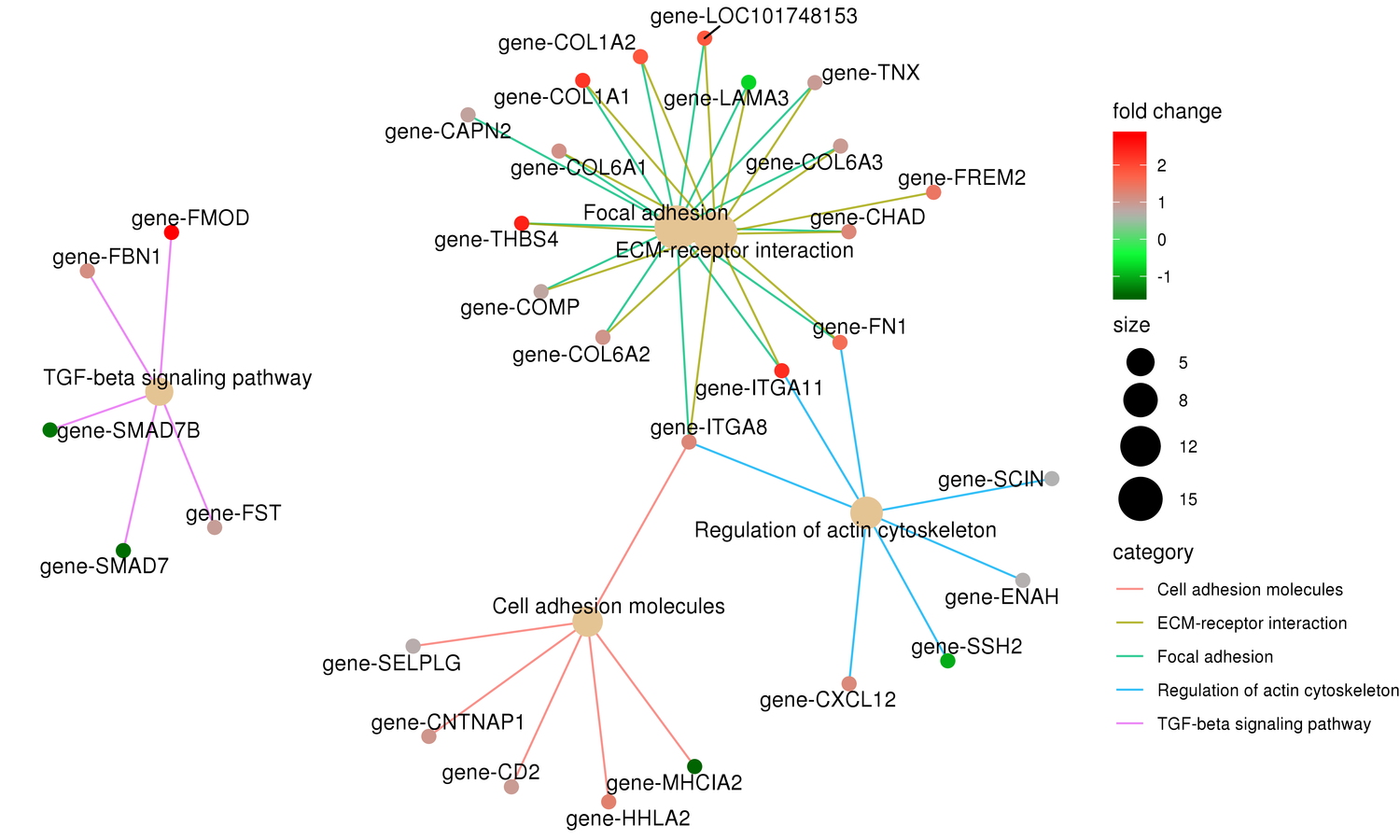

Supplement: S2 Fig — The color of the edge represents different pathways, and the color of the gene node represents the difference multiple. The larger the pathway node is, the more genes are enriched into the pathway. (PNG) [file pone.0326128.s002.png]
